# PARP14: A key ADP-ribosylating protein in host–virus interactions?

**DOI:** 10.1371/journal.ppat.1010535

**Published:** 2022-06-09

**Authors:** Srivatsan Parthasarathy, Anthony R. Fehr

**Affiliations:** Department of Molecular Biosciences, University of Kansas, Lawrence, Kansas, United States of America; University of Wisconsin-Madison, UNITED STATES

Over 300 posttranslational modifications (PTMs) are known to modify the functions of proteins by affecting processes ranging from activation, degradation, localization, secretion, recognition, and regulation [1]. One such PTM, ADP-ribosylation, can be defined as the transfer of a single ADP-ribose (Mono-ADP-ribosylation (MAR)) or multiple ADP-ribose (Poly-ADP-ribosylation (PAR)) units to target proteins utilizing nicotinamide adenine dinucleotide (NAD^+^) as the substrate. PARP14 is a MARylating enzyme that is implicated in a range of processes from tumorigenesis to DNA repair. Most notably, PARP14 is well known in the literature for promoting the anti-inflammatory interleukin (IL)-4–mediated signaling pathway by activating STAT6-dependent gene expression and inhibiting STAT-1–dependent gene expression. However, PARP14 expression is also induced by interferon (IFN), and it enhances host IFN responses to lipopolysaccharide (LPS), poly(I:C), and viral infection, indicating a role for PARP14 in restricting viral and bacterial infections. Despite these results, data supporting a significant role for PARP14 in the antiviral response are limited. More studies are needed to identify specific roles for PARP14 during viral infections, determine its targets following infection, and elucidate the mechanisms by which PARP14 modulates inflammatory pathways.

## What is PARP14?

**Fig 1:**
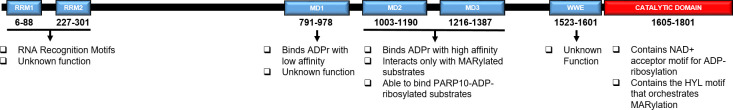
Domain architecture of PARP14 with known functions of each domain. MD, macrodomain; NAD, nicotinamide adenine dinucleotide; RRM, RNA recognition motif.

PARP enzymes are ADP-ribosyltransferases (ARTs) that transfer ADP-ribose from NAD^+^ onto a target protein or nucleic acid [2]. PARP14 is the largest PARP, consisting of 1,801 amino acids (Fig 1), and is restricted to Mono-ART activity. PARP14 is also know by other names like B Cell aggressive lymphoma 2 (BAL2) and Collaborator of STAT6 (CoaST-6). It belongs to a unique group of Macro-PARPs, which also includes PARP9 and PARP15 that contains multiple macrodomains (MDs). These MDs are characterized by their ability to bind MARylated, but not PARylated proteins [3].

The PARP14 catalytic domain contains a NAD^+^ binding pocket with several key amino acid residues that catalyze the transfer of ADP-ribose subunit to the appropriate protein targets [4]. These catalytic residues include an HYL amino acid motif, where the leucine residue replaces a key glutamate residue required for PARylating activity, which restricts PARP14 to mono-ART activity [5].

Apart from these 2 domains, PARP14 also contains RNA recognition motifs (RRM) and a WWE domain (Fig 1). There are 2 contiguous RRMs present in the amino terminus of the protein; however, their binding targets are yet to be discovered. WWE domains are characterized by the presence of a Trp-Trp-Glu region, which is important for PARP14 protein structure stabilization [4]. WWE domains can bind to ADP-Ribose derivatives, including iso-ADP-ribose, a molecule specifically found in PAR [6], and promote interactions with ubiquitinated proteins [7]. However, the PARP14 WWE domain was unable to bind to any ADP-ribose derivatives and whether it interacts with iso-ADP ribose or ubiquitinated targets is unknown [8].

## What are the known functions of PARP14?

**Fig 2:**
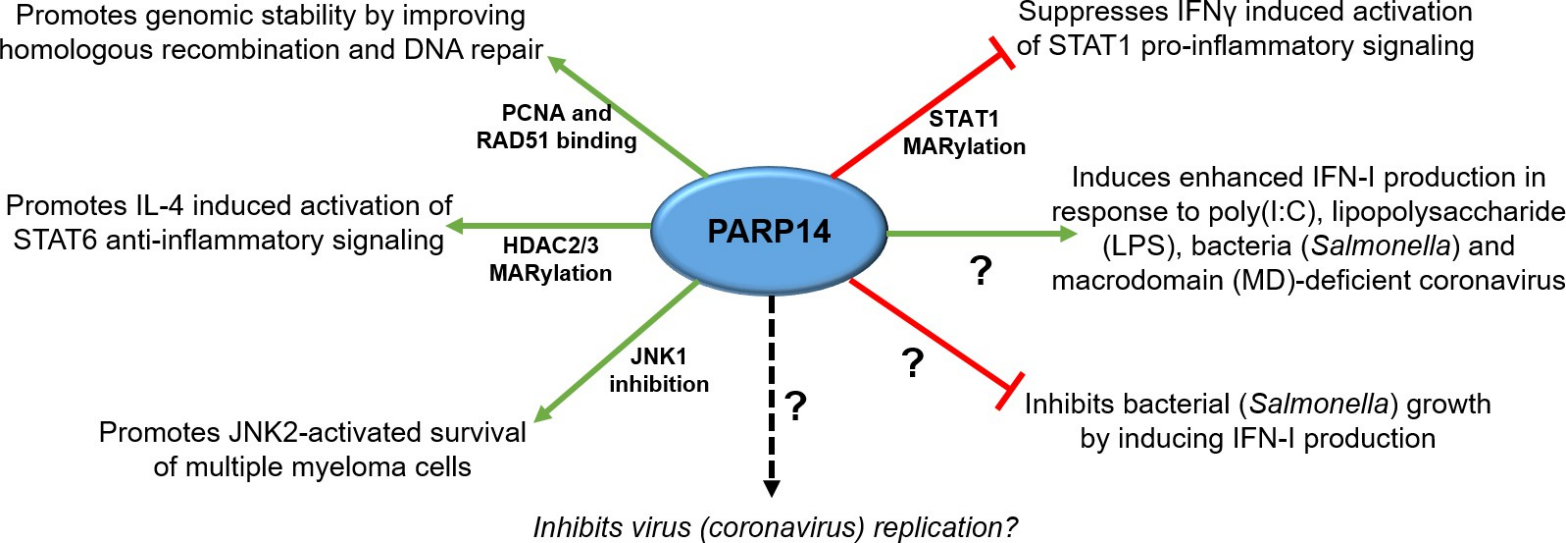
Known functions of PARP14 and their mechanisms of action. IFN, interferon; IL, interleukin; LPS, lipopolysaccharide; MD, macrodomain.

PARP14 affects several processes including cell differentiation, DNA repair, transcriptional control, and inflammatory signaling pathways (Fig 2). PARP14 selectively binds to STAT6 and promotes the expression of IL-4–regulated genes [9]. Since this original discovery, PARP14’s ability to regulate IL-4 signaling pathway has been implicated in several diseases. In one study, PARP14 promoted IL-4–mediated protection of B cells against apoptosis and IL-4–mediated expression of other B cell survival factors like Pim-1, 2, and Bcl2 [10], possibly explaining the overexpression of PARP14 in B cell aggressive lymphoma (BAL) [9]. PARP14 activation of STAT6-dependent gene expression also led to increased T_H_2 cell differentiation and allergic reactions, manifesting in the form of allergic airway disease [11]. Importantly, allergic responses could be partially mitigated by a highly specific PARP14 inhibitor [12].

PARP14 is also implicated in functions independent of STAT6. Recently, Barbarulo and colleagues found that PARP14 expression was induced by the presence of JNK2, which promoted cell growth of cancer cells in multiple myeloma [13]. Using shRNA knockdown of PARP14 and ectopic expression of truncated PARP14, they demonstrated that PARP14 reduced the activity of JNK1 via direct binding and prevented alleviation of carcinogenesis via apoptosis in cellulo [13]. In contrast, PARP14 also alleviated cancer by promoting genomic stability by improving homologous recombination and DNA repair in HeLa cells [14]. MD2 of PARP14 bound to RAD51 and PCNA components of the DNA replication machinery and promoted efficient DNA replication in DNA break sites [14]. A recent report found that PARP14 was required for cell cycle progression by regulating cyclin D1 expression [15]. This study found that cancer cell lines like RPE-1 and MCF-7 are arrested in G1 phase of cell cycle upon PARP14 depletion. Given the involvement of PARP14 in these different pathologies and cellular processes, it is critical to understand how PARP14 mediates these effects and is a major topic of ongoing research in various fields.

## How does PARP14 regulate inflammation?

As mentioned above, PARP14 regulates IL-4–dependent [16] transcriptional activation of STAT6 [17] via promoter interaction and MARylation of HDAC2/3. HDAC2/3 MARylation acts to dissociate these proteins from the promoter region, ultimately increasing acetylation of histones in promoter regions, allowing target gene activation [18]. In addition, Iwata and colleagues found that, in M1 macrophages, PARP14 suppressed IFNγ-induced inflammatory response, while inducing IL4-dependent anti-inflammatory effects. Mechanistically, PARP14 MARylated STAT1 at sites proximal to it phosphorylation sites, which likely affected STAT1 phosphorylation and suppressed its pro-inflammatory function [19]. These findings were supported by enhanced arterial lesion development and atherogenesis in PARP14-deficient mice. These results suggest that PARP14 could be a therapeutic target for attenuating inflammatory disorders [19].

## Does PARP14 regulate signaling in response to pathogens?

Recently, we and others found that PARP14 enhances the type I interferon (IFN-I) response to pathogens [20]. Caprara and colleagues found that PARP14 up-regulated IFN-I in response to LPS in RAW 264.7 macrophages and primary bone-marrow derived M0 macrophages (BMDMs). In response to LPS, PARP14 activated the expression of several IRF3 target genes, including IFN-β, but not the expression, phosphorylation, or localization of IRF3 itself. Interestingly, pol II recruitment to the nucleus and H3K27 acetylation was reduced in IRF3 promoter regions in the absence of PARP14, suggesting again that it may be altering the function of HDACs. In addition to LPS, PARP14 altered the cellular response to a bacterial infection, as *Salmonella typhimurium* bacterial load was elevated and IRF-3 target gene expression was reduced in PARP14-depleted cells [20]. This finding suggests PARP14 could play a regulatory role in immune pathways in response to pathogens.

PARP14 also induces IFN-I following poly(I:C) treatment, a double-stranded RNA mimic, and virus infection. In the A549 lung epithelial cell line, we found that deletion of PARP14 reduces IFN-I in response to poly(I:C) [21]. To demonstrate the role of PARP14 in virus-induced IFN induction we utilized a MD-deficient murine coronavirus, murine hepatitis virus (MHV), a model coronavirus. Viral MDs possess ADP-ribosylhydrolase activity and counter PARP activity. A recombinant MHV MD mutant virus (N1347A) enhances the IFN response in BMDMs compared to wild-type virus, indicating that ADP-ribosylation promotes IFN induction following MHV infection. This increased IFNβ production was completely ablated in the absence of PARP14, again demonstrating that it has a critical role in IFNβ induction [21]. However, the impact of PARP14 on STAT1 signaling and ISG expression in M0 macrophages or epithelial cells, as demonstrated in M1 macrophages following IFNγ treatment [19], has not been addressed. Thus further research on this topic is necessary. Regardless, the clear role of PARP14 in inducing IFNβ indicates that it may play a critical role in host–virus interactions.

## Does PARP14 restrict viral replication and/or pathogenesis?

Several pieces of circumstantial evidence indicate that PARP14 may be involved in the repression of viral infections. First, PARP14 expression is stimulated in a variety of viral infections such as Chikungunya virus [22] and β-coronaviruses like MHV and severe acute respiratory syndrome coronavirus 2 (SARS-CoV-2) [21,23]. Second, PARP14 was also identified as one of the 5 human PARP genes that has evolved under positive selection, a trait common to genes that are usually involved in immune response to pathogens [24]. Third, we demonstrated that siRNA knockdown of PARP14 mildly enhanced the replication of MHV N1347A, but not WT virus. However, PARP14 knockout BMDMs did not show enhanced MD mutant virus replication, calling into question the role of PARP14 in restricting the replication of MHV [21]. Finally, one might predict that PARP14 could reduce immune pathology during infection in vivo owing to its ability repress pro-inflammatory signaling via STAT6 [25]. The combination of these factors strongly indicates that PARP14 could be an antiviral factor. However, there is no convincing evidence where knockdown, knockout, or even overexpression of PARP14 results in altered virus replication or pathogenesis in cell culture or in vivo. The emergence of a potent and highly specific PARP14 inhibitor, RBN012759 [12], and other knockout cell culture and mouse models will be useful in identifying infections where viral replication, immune signaling pathways, and cell death pathways both in vitro and in vivo are modulated by PARP14. However, given PARP14’s effect on crucial cellular functions like DNA repair and the cell cycle, an inducible PARP14 knockout system in vivo and in vitro may be necessary to accurately assess the effect of PARP14 depletion on viral replication and pathogenesis. Since PARP14 is implicated in several diseases, including cancer, a full exploration of its potential role in countering virus infection is needed to establish a platform to determine its targets, elucidate the mechanisms by which PARP14 modulates inflammatory pathways, and develop novel therapies.
